# Class-B CpG-ODN Formulated With a Nanostructure Induces Type I Interferons-Dependent and CD4^+^ T Cell-Independent CD8^+^ T-Cell Response Against Unconjugated Protein Antigen

**DOI:** 10.3389/fimmu.2018.02319

**Published:** 2018-10-10

**Authors:** Ana L. Chiodetti, María F. Sánchez Vallecillo, Joseph S. Dolina, María I. Crespo, Constanza Marin, Stephen P. Schoenberger, Daniel A. Allemandi, Santiago D. Palma, María C. Pistoresi-Palencia, Gabriel Morón, Belkys A. Maletto

**Affiliations:** ^1^Departamento de Bioquímica Clínica, Facultad de Ciencias Químicas, Universidad Nacional de Córdoba, Córdoba, Argentina; ^2^Consejo Nacional de Investigaciones Científicas y Técnicas, Centro de Investigaciones en Bioquímica Clínica e Inmunología, Córdoba, Argentina; ^3^Division of Developmental Immunology, La Jolla Institute for Allergy and Immunology, La Jolla, CA, United States; ^4^Departamento de Farmacia, Facultad de Ciencias Químicas, Universidad Nacional de Córdoba, Córdoba, Argentina; ^5^Consejo Nacional de Investigaciones Científicas y Técnicas, Unidad de Tecnología Farmacéutica, Córdoba, Argentina

**Keywords:** CpG-ODN, nanostructure, vaccine, adjuvant, CD8^+^ T-cell response, type I interferons, ascorbyl palmitate ester

## Abstract

There is a need for new vaccine adjuvant strategies that offer both vigorous antibody and T-cell mediated protection to combat difficult intracellular pathogens and cancer. To this aim, we formulated class-B synthetic oligodeoxynucleotide containing unmethylated cytosine-guanine motifs (CpG-ODN) with a nanostructure (Coa-ASC16 or coagel) formed by self-assembly of 6-0-ascorbyl palmitate ester. Our previous results demonstrated that mice immunized with ovalbumin (OVA) and CpG-ODN formulated with Coa-ASC16 (OVA/CpG-ODN/Coa-ASC16) elicited strong antibodies (IgG1 and IgG2a) and Th1/Th17 cellular responses without toxic systemic effects. These responses were superior to those induced by a solution of OVA with CpG-ODN or OVA/CpG-ODN formulated with aluminum salts. In this study, we investigated the capacity of this adjuvant strategy (CpG-ODN/Coa-ASC16) to elicit CD8^+^ T-cell response and some of the underlying cellular and molecular mechanisms involved in adaptive response. We also analyzed whether this adjuvant strategy allows a switch from an immunization scheme of three-doses to one of single-dose. Our results demonstrated that vaccination with OVA/CpG-ODN/Coa-ASC16 elicited an antigen-specific long-lasting humoral response and importantly-high quality CD8^+^ T-cell immunity with a single-dose immunization. Moreover, Coa-ASC16 promoted co-uptake of OVA and CpG-ODN by dendritic cells. The CD8^+^ T-cell response induced by OVA/CpG-ODN/Coa-ASC16 was dependent of type I interferons and independent of CD4^+^ T-cells, and showed polyfunctionality and efficiency against an intracellular pathogen. Furthermore, the cellular and humoral responses elicited by the nanostructured formulation were IL-6-independent. This system provides a simple and inexpensive adjuvant strategy with great potential for future rationally designed vaccines.

## Introduction

Most current vaccines rely on antibody production for protection but fail to generate robust T-cell immunity crucial for combating intracellular pathogens and cancer (1, 2). To overcome this challenge, new adjuvant strategies are being developed worldwide in experimental models or in human clinical trials (3–5). Among them, there is a special interest on synthetic oligodeoxynucleotides containing unmethylated cytosine-guanine motifs (CpG-ODN), agonists of Toll-like receptor 9. The key features of CpG-ODN used as a vaccine adjuvant, in contrast to currently licensed adjuvants, include the ability to elicit antibody, Th1-like over a Th2-like CD4^+^ T-cell response and, but only under certain conditions, CD8^+^ T-cell immunity. Over the last decade many human clinical trials have been carried out with CpG-ODN, some of which are in phase III trials in the vaccine area (6–9). Most recently, CpG-ODN has been used as adjuvant in a vaccine (Heplisav-B) licensed by FDA, indicated for the prevention of infection caused by Hepatitis B Virus in adults 18 years of age and older (10). However, the use of free CpG-ODN still presents some limitations such as unfavorable pharmacokinetics/biodistribution patterns, high binding to plasma proteins, lack of specificity for target cells and poor cellular uptake that subsequently restricts its bioavailability (9, 11–13). Hence, there is great interest in developing efficient strategies to sort these difficulties and optimize the CpG-ODN immunostimulatory activity. To this end, multiple strategies such as nano/microparticles construed in a variety of ways using different materials and self-assembled DNA nanostructures have been explored. Although most of these formulations appeared promising, some of them also had some problems mainly related to manufacturing issues, such as the scaling-up of production, and toxicity associated with cationic materials (11, 12, 14, 15).

In order to optimize the adjuvant activity of CpG-ODN, we formulated it with a nanostructure (Coa-ASC16 or coagel) formed by self-assembly of 6-O-ascorbyl palmitate ester (ASC16). Our previous results demonstrated that the nanostructured formulation of ovalbumin (OVA) and CpG-ODN with Coa-ASC16 (OVA/CpG-ODN/Coa-ASC16) remarkably enhanced humoral (IgG1, IgG2a) and cellular (Th1 and Th17) responses in comparison to the soluble counterpart (OVA/CpG-ODN) under a three-dose immunization scheme. When compared the efficiency of CpG-ODN/Coa-ASC16 with CpG-ODN formulated in aluminum salts, we observed that the immunization with OVA/CpG-ODN/Coa-ASC16 was significantly more efficient than CpG-ODN/Al(OH)_3_ to induce specific humoral (IgG1 and IgG2a), Th1 and Th17 cellular immune responses. In addition, our preclinical systemic toxicology studies performed at days 21 and 197 after first immunization showed than CpG-ODN/Coa-ASC16 did not induce adverse biological effects (16).

**Figure 1:**
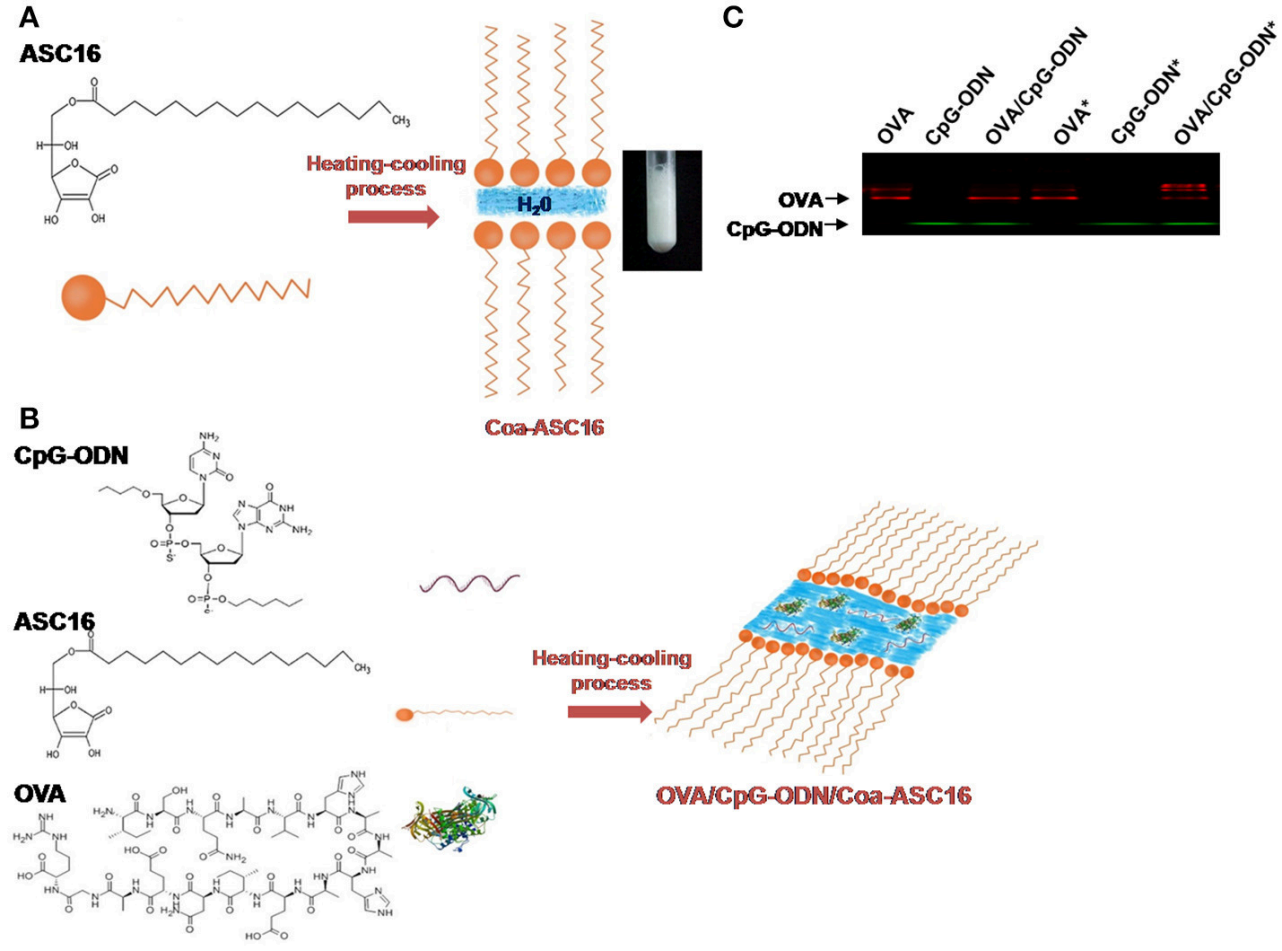
The formulation of OVA and CpG-ODN with the nanostructure. **(A)** Schematic representation of Coa-ASC16 and photographic image. **(B)** Schematic representation of OVA/CpG-ODN/Coa-ASC16. **(C)** Native-PAGE of formulations using IRDye® 680RD OVA and 5′ IRDye® 800CW CpG-ODN comparing non-treated and heat-treated samples (marked with asterisk).

ASC16 is an amphiphilic molecule composed of an ascorbic acid polar headgroup attached to a palmitic acid nonpolar hydrocarbon chain. When an aqueous dispersion of ASC16 is heated above the critical micelle temperature, at which the solubility reaches the critical micelle concentration, aggregates form a gel phase. Upon cooling below the critical micelle temperature, Coa-ASC16 is formed. Our previous studies showed that Coa-ASC16 is a hydrated crystalline phase and their lamellar structure produces at least one highly ordered dimension, so they exhibit sharp X-ray diffraction patterns and optical birefringence. The surfactant hydrocarbon chains have limited freedom of motion, with an interlayer distance of about 10 Å, water occupies the space between the surfactant lamellae (17). After adding CpG-ODN and OVA (both hydrophilic components), the study of behavior of the H_2_O interlayers suggests that they are situated in the aqueous interlamellar domain (18). A schematic picture of this system is shown in Figures 1A,B. Coa-ASC16 has many advantages that make it a very attractive platform for biomedical use: (i) it is formed by two biodegradable components (ascorbic acid and palmitic acid), (ii) ASC16 is listed as a Generally Recognized as Safe substance, and (iii) it is easy to prepare and inexpensive.

The purpose of the present study was to investigate the capacity of this new adjuvant strategy (CpG-ODN/Coa-ASC16) or free CpG-ODN to elicit CD8^+^ T-cell response and some of the underlying cellular and molecular mechanisms involved in adaptive response. We also analyzed whether this adjuvant strategy allows a switch from an immunization scheme of three-doses to one of single-dose. Our results demonstrated that vaccination with OVA/CpG-ODN/Coa-ASC16 induced long-lasting humoral response and importantly-high quality CD8^+^ T-cell immunity with a single-dose immunization. The CD8^+^ T-cell response induced by OVA/CpG-ODN/Coa-ASC16 was dependent of type I interferons (IFN-I) and independent of CD4^+^ T cells, and showed polyfunctionality and efficiency against an intracellular pathogen. Furthermore, the cellular and humoral responses elicited by the nanostructured formulation were IL-6-independent. Therefore, the present work helps to understand the mechanism of action of this new adjuvant strategy and extends its potential use for future rationally designed vaccines.

## Materials and methods

### Mice

Wild-type (WT) C57BL/6 mice were purchased from Fundación Facultad de Ciencias Veterinarias (Universidad Nacional de La Plata, La Plata, Argentina), *Il6*^−/−^ and *Cd8a*^−/−^ mice from Jackson Laboratory (Bar Harbor, ME, USA). *Ifnar1*^−/−^ mice were kindly provided by Dr. M. Albert (Institut Pasteur, Paris, France). All mice were bred in our animal facility in accordance with the standards of the Guide to the Care and Use of Experimental Animals, published by the Canadian Council on Animal Care; with the assurance number A5802-01 delivered by the Office of Laboratory Animal Welfare (NIH). The experiments were conducted on 8–12 weeks-old female mice following protocol approved by the Institutional Animal Experimentation Committee, Facultad de Ciencias Químicas, Universidad Nacional de Córdoba in Argentina (# 907/2015) and by the Institutional Animal Care and Use Committee from USA (# AP007-SPS1-0116).

### Reagents

OVA was from Worthignton Biochemical Corporation (Lakewood, NJ, USA). A Detoxi-Gel™ Endotoxin Removing column (Thermo Fisher Scientific, Buenos Aires, Argentina) was used to reduce the endotoxin level of the OVA stock solution up to <1 EU/ml (Endosafe Test, Charles River, Wilmington, MA, USA). Class-B CpG-ODN 1826 [5′-TCCATGACGTTCCTGACGTT-3′] with total phosphorothioate-modified was used (Operon Technologies, Alameda, CA, USA). The endotoxin content in CpG-ODN after reconstitution, determined by a standard Limulus amebocyte lysate assay (BioWhittaker Inc., Walkersville, MD, USA) was <1 EU/ml. 5′ IRDye® 800CW CpG-ODN 1826 was synthesized by IDT (San Diego, CA, USA) and OVA was labeled with IRDye® 680RD using the IRDye® 680 Protein Labeling Kit-high MW (LI-COR Biosciences, Lincoln, NE, USA) according to manufacturer's instructions. Alexa Fluor 647® OVA was purchased from Thermo Fisher Scientific and 5′ Alexa Fluor 488® CpG-ODN 1826 from IDT. ASC16 was from Fluka Analytical (Milan, Italy) and the H-2K^b^ restricted OVA_257−264_ (SIINFEKL) peptide from Invivogen (San Diego, CA, USA). Sterile apyrogenic 5% dextrose solution was from Roux-Ocefa (Buenos Aires, Argentina) and Complete Freund's Adjuvant (CFA) from Sigma-Aldrich (Buenos Aires, Argentina).

### Preparation of Coa-ASC16-based formulations

OVA and/or CpG-ODN were added to a dispersion of 2% (w/v) ASC16 in 5% dextrose solution, heated up to 72°C for 15 min and then left to reach room temperature as described previously (16).

### Immunization

Mice were subcutaneously injected with OVA and CpG-ODN in solution (OVA/CpG-ODN), OVA and CpG-ODN formulated with the Coa-ASC16 (OVA/CpG-ODN/Coa-ASC16), OVA formulated with Coa-ASC16 (OVA/Coa-ASC16) or CpG-ODN formulated with Coa-ASC16 (CpG-ODN/Coa-ASC16). Each mouse was immunized with an entire volume of 250 μl equally distributed in 5 sites: the tail base, back and neck region and both hind limbs. CpG-ODN was administered at 75 μg/mouse/dose and OVA at 6 μg/mouse/dose in all experiments except for the one shown in Figure **3** where the dose of OVA was reduced to 2 μg/mouse/dose. Two different immunization schemes were used: (1) mice were immunized on days 0, 7, and 14 and (2) mice were immunized once at day 0. At different time post immunization, blood was collected in heparinized capillary tubes to measure the anti-OVA antibody titers in plasma.

An additional group was immunized with OVA formulated with CFA (OVA/CFA) on days 0, 15 and 30.

In some experiments, mice were intraperitoneally injected with anti-CD4 (GK1.5) antibody on days −2, −1, 0, and 2 of immunization (100 μg/mouse/dose) for CD4^+^ T-cell depletion or isotype control (IgG). Control of depletion (see Supplementary Figure 1).

### Native-page

Formulations were prepared using CpG-ODN and OVA coupled to near-infrared dye at the final concentrations used to immunize. The CpG-ODN solution was used in a proportion of 1/180 5′ IRDye® 800CW CpG-ODN/not labeled CpG-ODN. Before loading, half of the samples were pre-heated at 72°C for 15 min and then cooled down to room temperature. Samples were loaded in 5% (v/v) glycerol and resolved in a 20% polyacrylamide gel containing no sodium dodecyl sulfate. The gel was scanned using an Odyssey Infrared Imaging System (LI-COR Biosciences).

### *In vivo* cytotoxicity assay

Splenocytes of non-immunized syngeneic mice were prepared. Half of the cells were incubated with 10 μg/mL of SIINFEKL peptide at 37°C for 30 min, then stained with 1.5 μM CFSE (Thermo Fisher Scientific). The remaining cells were stained with 0.15 μM CFSE. Immunized and non-immunized (control) mice were intravenously injected with a 1:1 mixture of these cells (10 × 10^6^ of each/mouse). Splenocytes of recipient mice were collected 24 h after transfer, and CFSE^+^ cells were measured by flow cytometry. Cytotoxicity is expressed by percentage of lysis calculated as [1–(r_control_-r_immune_)] × 100, where *r* is given by the expression of %CFSE^low^/%CFSE^high^ cells from non-immunized and immunized mice, respectively. This assay was performed in WT, *Il6*^−/−^*, Cd8a*^−/−^, and *Ifnar1*^−/−^ recipient mice using target cells from each mouse strain respectively.

### Splenocytes preparation and cytokines quantification

To prepare splenocytes, single cell suspensions of spleen were treated with lysing buffer (Sigma-Aldrich).

Splenocyte cultures was performed in a 96-well U-bottom plate (Greiner Bio One, Frickenhausen, Germany) at 37°C with 5% CO_2_ in GIBCO®RPMI 1640 medium (Thermo Fisher Scientific) supplemented with 10% FBS (NATOCOR, Carlos Paz, Argentina), 1% GIBCO® GlutaMAX, 100 U/ml penicillin, 100 μg/ml streptomycin (all from Thermo Fisher Scientific) and 50 μM 2-mercaptoethanol (Sigma-Aldrich).

To determine IFN-γ concentration in supernatants, splenocytes (1 × 10^6^ cell/well) were stimulated for 72 h at 37°C with SIINFEKL peptide (1 μg/ml) or OVA (100 μg/ml) and IFN-γ measured by ELISA (IFN-γ ELISA MAX^TM^kit, Biolegend, San Diego, CA, USA). The supernatant IFN-γ concentration was calculated after subtraction of background response (cells incubated with media).

To determine intracellular cytokines, splenocytes (3 × 10^6^ cells/well) were stimulated for 5 h with SIINFEKL peptide (2 μg/ml) in the presence of GolgiStop (0.7 μl/ml) and GolgiPlug (1 μl/ml) (BD Bioscience, San Diego, CA, USA). In some experiments, anti-CD107a (ID4B) antibody was added during the incubation with the peptide/GolgiStop/GolgiPlug mix.

### Flow cytometry

Combinations of fluorochrome-labeled antibodies from eBiosecience, Biolegend, BD Biosciences or Tonbo Biosciences (San Diego, CA, USA) were used. To determine intracellular cytokines, cells were surface stained with anti-CD8α (53-6.7) and -TCRβ (H57-597) antibodies. Then, fixed/permeabilized with Cytofix/Cytoperm™ Plus kit (BD Bioscience) according to manufacturer's instructions and stained for cytokines using anti-TNF-α (MP6-XT22), -IFN-γ (XMG1.2) and -IL-2 (JE56-5H4) antibodies. The co-expression of the surface CD107a protein and the cytokines IFN-γ, TNF-α, IL-2 were determined after subtraction of background response (cells incubated with media) using FlowJo Boolean gating platform. To determine SIINFEKL-K^b^ tetramer^+^ cells, splenocytes were incubated for 15 min at 4°C with anti-TCRβ (H57-597), -CD4 (RM4-5) and -CD8α (53-6.7) antibodies and SIINFEKL-K^b^ tetramer-PE (NIH Tetramer Core Facility at Emory University, Atlanta, GA, USA). The cells were collected on a FACSCanto II cytometer and the data were processed with FlowJo software (Tree Star, Ashland, OR, USA).

### Anti-OVA antibody titers

Antibody titers were measured by ELISA flowing a previously described protocol (16). HRP-conjugated anti-mouse IgG (Sigma-Aldrich), IgG1 (X56) and IgG2a/c (R19-15) (both from BD Bioscience) were used as detection antibodies. Titer was considered to be the reciprocal of the last plasma dilution that yielded a value of optical density (OD) at 490 nm greater than that of twice the mean value of reagent blank. The plasmas from non-immunized mice were not reactive to OVA.

### Determination of antibody avidity

96-well half area high binding plates (Greiner Bio One) were coated with OVA (1 μg/well) in 0.1 M sodium carbonate-bicarbonate buffer (pH 9.6) and incubated overnight at 4°C. Plates were washed with 0.05% Tween®20-PBS and blocked with 0.5% gelatin-PBS. Then, washed and incubated for 1 h at 37°C with plasma samples at a dilution that gave an OD value at 490 nm between 1.0 and 2.0 in the standard ELISA. Following another washing step, 50 μl of increasing concentrations (0, 0.5, 1.0, 1.5, 2.0, and 2.5 M) of potassium thiocyanate (KSCN) were added to each row of the plate for 15 min. Plates were washed and incubated with anti-mouse HPR-conjugated IgG antibody (Sigma-Aldrich). Plates were developed by adding substrate (o-phenylenediamine and H_2_O_2_) and OD determined at 490 nm. The OD values in the KSCN-treated wells were expressed as a percentage of the untreated reference well as previously described (19).

### *In vivo* uptake of OVA and CpG-ODN

Mice were subcutaneously immunized in both hind limbs with OVA/CpG-ODN or OVA/CpG-ODN/Coa-ASC16 (1.2 μg OVA and 15 μg CpG-ODN/50 μl/site) using Alexa Fluor 647® OVA and a 50:50 mix of 5′ Alexa Fluor 488® CpG-ODN and unlabeled CpG-ODN. Seventy-two h later, inguinal lymph nodes (LN) were harvested from which a single cell suspension was obtained after collagenase D (0.5 mg/ml)/DNase I (0.2 mg/ml) (Sigma-Aldrich) treatment. Cells were pre-incubated with anti-CD16/32 (2.4G2) and subsequently stained at 4°C for 15 min with anti-CD11c (N418) antibody (Biolegend) for flow cytometry analysis.

### Infection

*Listeria monocytogenes* 10403s strain with OVA construct (*Lm-*OVA) and attenuated *Lm*-OVA (Δ*actA Lm-*OVA) were kindly provided by Dr. Daniel Portnoy (University of California, Berkeley, CA, USA). Mice were intravenously infected with 5 × 10^3^ Colony Forming Units (CFU) of Δ*actA Lm-*OVA or 1 × 10^5^ CFU of *Lm-*OVA. For bacterial load quantification, livers were homogenized in 0.2% Nonidet P-40 (Sigma-Aldrich)-PBS and serial 10-fold dilutions were plated on BHI agar (BD Biosciences) containing 200 μg/ml streptomycin. CFU were counted after growth for 24 h at 37°C.

### Statistical analysis

Data were analyzed using the GraphPad Prism5® (GraphPad Software, San Diego, CA, USA). In experiments with multiple groups of mice, statistical differences between treatment groups were compared using ANOVA and Bonferroni post test for multiple comparisons. For comparisons between two treatment groups, unpaired Student's *t* test was used. All data were considered statistically significant if *P* values were <0.05.

## Results

### The formulation of OVA and CpG-ODN with the nanostructure

Coa-ASC16-based scaffolding containing OVA and CpG-ODN is obtained after a heating-cooling process of a mix of three well-defined components (OVA, CpG-ODN, and ASC16) (Figure 1B). To test whether the manufacturing process could promote interactions between the OVA and CpG-ODN, solutions of OVA, CpG-ODN, or OVA/CpG-ODN were heated or left unheated and resolved by Native-PAGE after reaching room temperature. As shown in Figure 1C, there was no aggregate found between the OVA and the CpG-ODN after the heating-cooling process.

### Formulation of OVA/CpG-ODN with Coa-ASC16 optimizes humoral and CD8^+^ T-cell responses independently of IL-6

**Figure 2:**
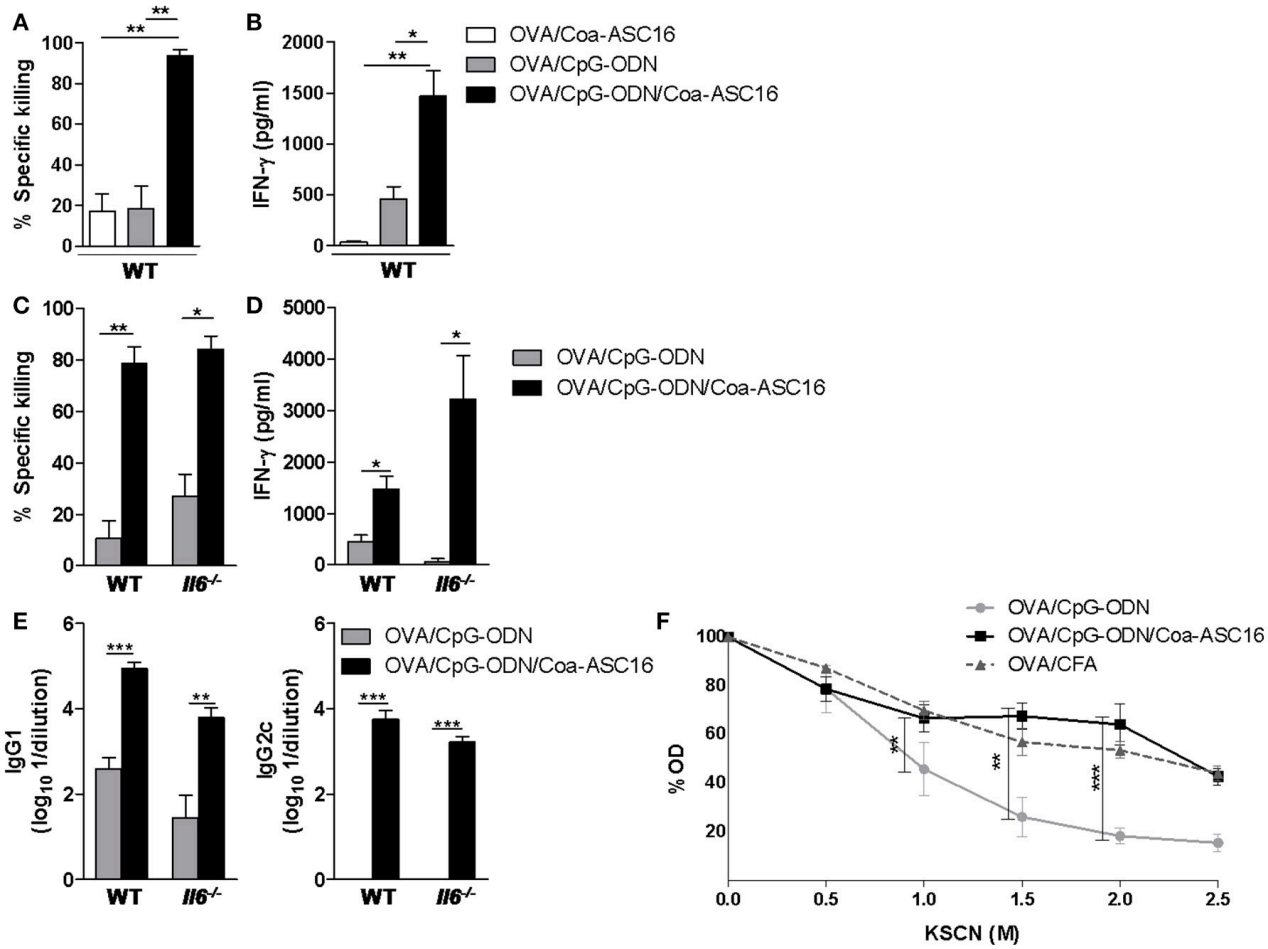
Formulation of OVA/CpG-ODN with Coa-ASC16 optimizes humoral and CD8^+^ T-cell responses independently of IL-6. WT or *Il6*^−/−^ mice were immunized with OVA/Coa-ASC16, OVA/CpG-ODN, OVA/CpG-ODN/Coa-ASC16 (on days 0, 7, and 14) or OVA/CFA (days 0, 15, and 30). **(A,C)** *In vivo* killing assay and **(B,D)** *ex vivo* IFN-γ secretion by splenocytes after stimulation with SIINFEKL peptide determined by ELISA on day 21. **(E)** Titers of OVA-specific IgG1 and IgG2c in plasma determined by ELISA on day 21. **(F)** WT mice. Avidity OVA-specific IgG in plasma determined by ELISA using KSCN elution seven days after the last immunization. The data show the mean ± SEM of individual values (3-4 mice/treatment group in each experiment) and are representative of two independent experiments performed. ^*^p < 0.05, ^**^p < 0.01, ^***^p < 0.001.

We have previously shown that OVA/CpG-ODN/Coa-ASC16 elicits Th1 cellular response (16), suggesting that it could also induce CD8^+^ T-cell response. To test whether the nanostructured formulation was able to induce OVA-specific CD8^+^ T-cell responses, mice were immunized with a three-dose schedule (days 0, 7, and 14) with OVA/Coa-ASC16, OVA/CpG-ODN, or OVA/CpG-ODN/Coa-ASC16. On day 21, *in vivo* killing assays were performed. Notably, mice immunized with OVA/CpG-ODN/Coa-ASC16 showed a superior cytotoxic activity than mice immunized with OVA/Coa-ASC16 or OVA/CpG-ODN (Figure 2A). Apart from direct cytolysis mechanisms, the CD8^+^ T-cells can also orchestrate a rapid host protection by crucial cytokines secretion for the activation of both innate and adaptive immune system (20, 21). In this regard, splenocytes from mice immunized with OVA/CpG-ODN/Coa-ASC16 showed higher IFN-γ secretion compared to those from mice immunized with OVA/Coa-ASC16 or OVA/CpG-ODN (Figure 2B).

Among other cytokines, IL-6 has been widely described as a promoter of the development of cytotoxic CD8^+^ T-cell (22) and antibody immunity in different adjuvant strategies (23–30). Since Coa-ASC16 enhances the CpG-ODN-induced humoral response (16) and that Coa-ASC16 alone (without antigen or CpG-ODN) is sensed by the innate immune system with a consequent local production of high amounts of IL-6 (31), we inquired whether this cytokine played a role in our model. To this end, we compared the antigen-specific immune response elicited by immunization with OVA/CpG-ODN/Coa-ASC16 in *Il6*^−/−^ vs. WT mice. The positive effects on CD8^+^ T-cell and humoral responses induced by Coa-ASC16 were not affected by the absence of IL-6 (Figures 2C–E).

Our previous studies have shown that the magnitude of OVA-specific humoral (IgG1, IgG2a) immune response from mice immunized with OVA/CpG-ODN/Coa-ASC16 is dramatically superior to that from mice immunized with OVA/CpG-ODN in solution (16). Here, we tested whether the formulation of the CpG-ODN with Coa-ASC16 had any impact on the quality of the humoral response. For that, we measured the avidity of the antibodies elicited by immunization with OVA/CpG-ODN, OVA/CpG-ODN/Coa-ASC16 and compared them with those generated by OVA/CFA as a model adjuvant system. Notably, the formulation of CpG-ODN with Coa-ASC16 improved the antibodies avidity up to a level comparable to those exerted by immunization with OVA/CFA (Figure 2F).

**Figure 3:**
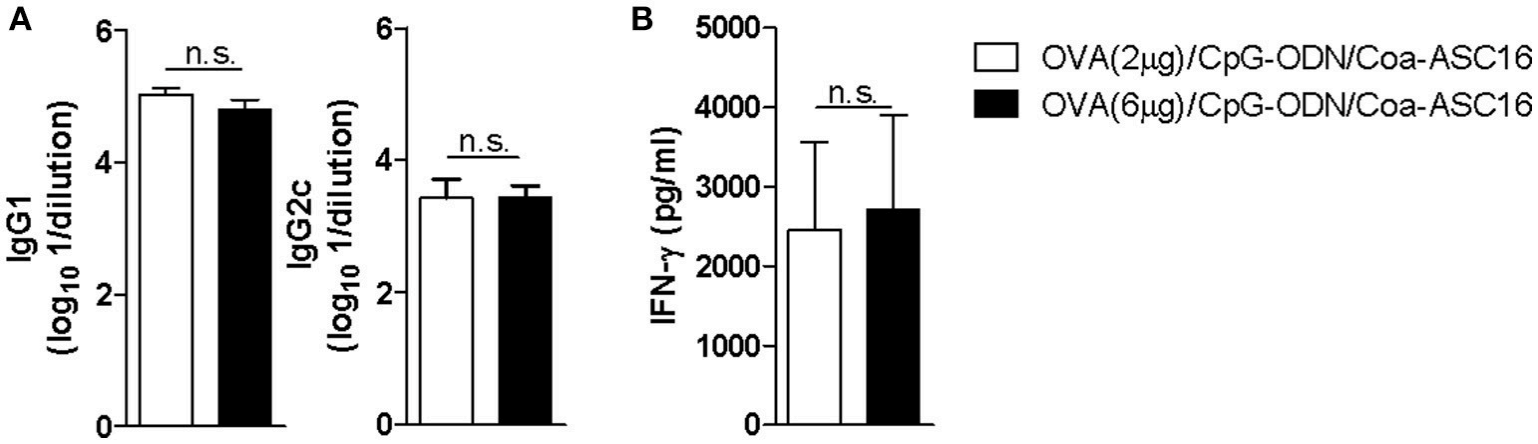
The nanostructured formulation allows antigen dose-sparing without compromise the resulting antigen-specific immune response. C57BL/6 mice were immunized on day 0, 7, and 14 with OVA (2 μg)/CpG-ODN/Coa-ASC16 or OVA (6 μg)/CpG-ODN/Coa-ASC16. Seven days after the last immunization, plasma and spleen were obtained. **(A)** Titers of OVA-specific IgG1 and IgG2c in plasma determined by ELISA. **(B)** *Ex vivo* IFN-γ secretion by splenocytes after stimulation with OVA determined by ELISA. The data show the mean ± SEM of individual values (4 mice/treatment group in each experiment) and are representative of two independent experiments performed. n.s., not significant.

In addition, we showed that the nanostructured formulation allows antigen dose-sparing without significantly affecting the antibody or Th1 cellular responses induced by vaccination (Figure 3). This characteristic is particularly important in cases were antigens are difficult to obtain or require high-cost manufacturing processes.

Together, these data demonstrated that Coa-ASC16 improves adjuvant effect of CpG-ODN with regard to the antibody avidity and CD8^+^ T-cell response. The humoral and cellular responses elicited by the nanostructured formulation not require IL-6 signaling.

### A single-dose of the nanostructured formulation is sufficient to induce robust humoral and CD8^+^ T-cell immunity

**Figure 4:**
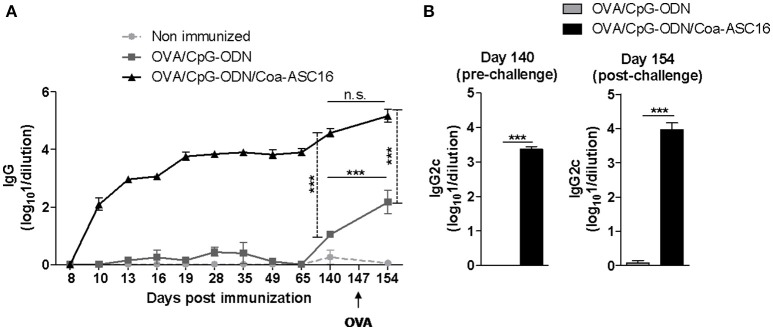
The nanostructured formulation elicits long-lasting humoral response with a single-dose immunization. C57BL/6 mice were immunized on day 0 with OVA/CpG-ODN or OVA/CpG-ODN/Coa-ASC16. On day 147 after immunization, mice were intraperitoneally challenged with OVA. Plasma samples were collected at different time points for determination of OVA-specific antibodies titers by ELISA. **(A)** Kinetics in plasma of OVA-specific IgG. **(B)** OVA-specific IgG2c on days 140 (pre-challenge) and 154 (post-challenge) after immunization. The data show the mean ± SEM of individual values (4 mice/treatment group in each experiment) and are representative of two independent experiments performed. ^***^p < 0.001, n.s., not significant.

**Figure 5:**
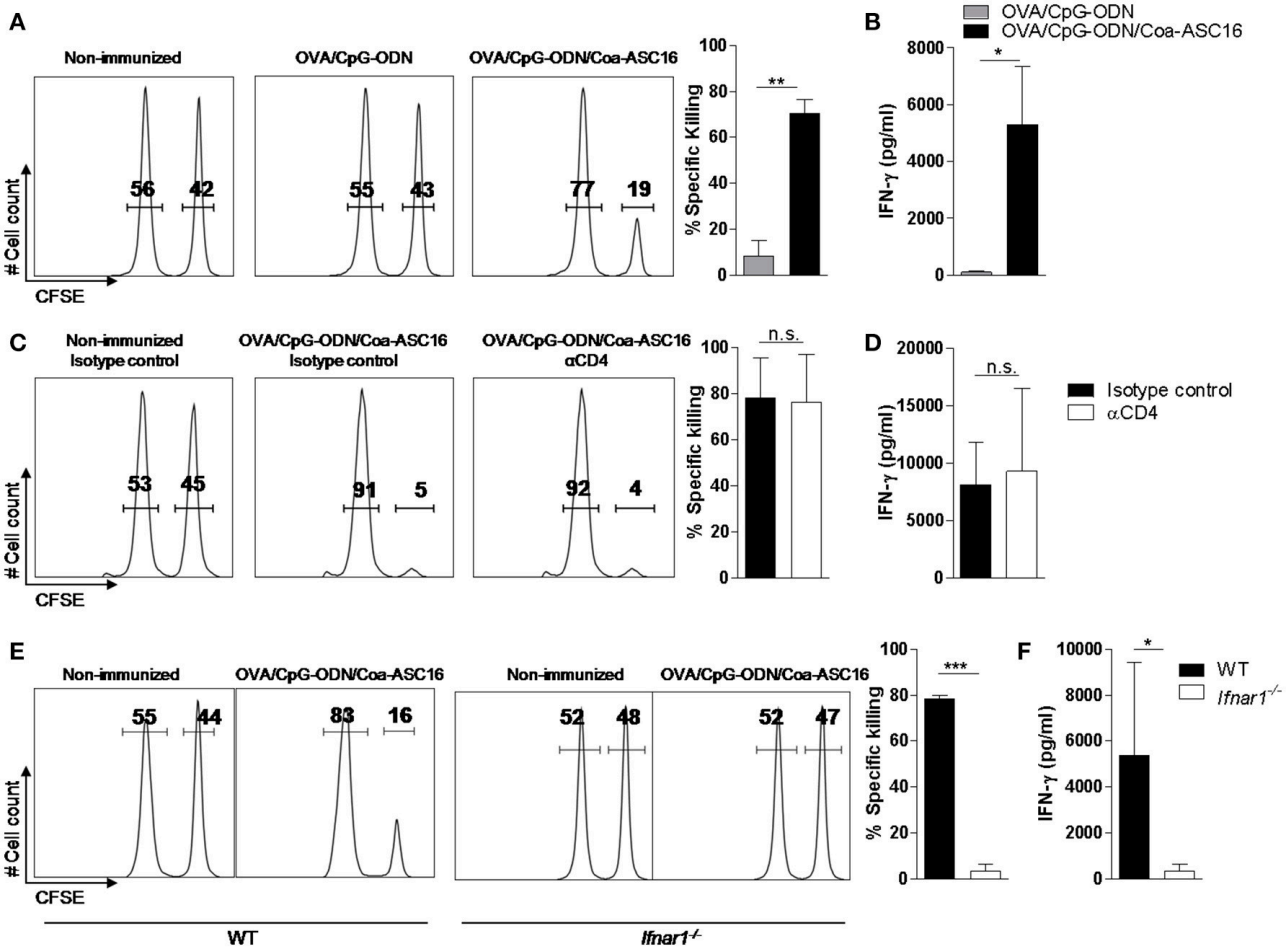
CD8^+^ T-cell response elicited by the nanostructured formulation is CD4^+^ T-cell independent and IFN-I dependent. WT **(A–D)** or *Ifnar1*^−/−^ **(E–F)** mice were immunized with a single-dose (day 0) of the indicated formulations. **(A,C,E)** *In vivo* killing assay and **(B**, **D** and **F)** *ex vivo* IFN-γ secretion by splenocytes after stimulation with SIINFEKL peptide determined by ELISA on day 7. In **C** and **D**, mice were treated on days −2, −1, 0, and 2 with anti-CD4 or isotype control (IgG) antibodies. The data show the mean ± SEM of individual values (3-4 mice/treatment group in each experiment) and are representative of two independent experiments performed. n.s., not significant, ^*^p < 0.05, ^**^p < 0.01, ^***^p < 0.001.

Three-dose regimens for vaccines are expensive and difficult to complete. Therefore, we asked whether a single-dose immunization was sufficient to induce an antigen-specific immune response. To this end, mice were immunized only at day 0. A single-dose with OVA/CpG-ODN/Coa-ASC16 elicited early seroconversion while OVA/CpG-ODN failed to generate OVA-specific IgG. In addition, we measured OVA-specific IgG after intraperitoneal challenge with OVA at day 147 post-immunization. This secondary contact with the antigen showed no significant effect on the humoral response induced by OVA/CpG-ODN/Coa-ASC16. However, mice immunized with OVA/CpG-ODN were able to seroconvert after secondary challenge but were never able to induce IgG2c subtype (associated with Th1-biased response) (Figures 4A,B). In addition, we analyzed the CD8^+^ T-cell response at day 7 after immunization. Mice immunized with OVA/CpG-ODN/Coa-ASC16 showed a cytotoxic activity and IFN-γ production comparable with the one obtained with a three-immunization schedule while mice immunized with OVA/CpG-ODN failed to elicit any CD8^+^ T-cell response (Figures 2A,B, and 5A,B). A supplementary *in vivo* killing assay was performed in *Cd8a*^−/−^ mice to demonstrate that the cellular lysis observed in our experimental model was exclusively carried out by CD8^+^ T-cells (Supplementary Figure 2). Therefore, our results demonstrate that vaccination with nanostructured formulation is able to induce a long-lasting antibodies and robust CD8^+^ T-cell responses with a single-dose. We next focus our efforts on the study of CD8^+^ T-cell response.

### CD8^+^ T-cell response is CD4^+^ T-cell independent

CD4^+^ T-cell help is generally required for the generation of CD8^+^ T-cell response. However, it has been reported that CpG-ODN can bypass this need and generate a help-independent CD8^+^ T-cell response (32, 33). To address the question of whether our adjuvant strategy had the same ability, we studied the CD8^+^ T-cell response in OVA/CpG-ODN/Coa-ASC16 immunized mice depleted from CD4^+^ T-cells. Our findings indicate that this response is help-independent (Figures 5C,D).

### IFN-I signaling is essential for the induction of CD8^+^ T-cell response

IFN-I are positive regulators of CD8^+^ T-cell response through multiple direct and indirect mechanisms (34, 35), we analyzed the CD8^+^ T-cell response elicited by the immunization with OVA/CpG-ODN/Coa-ASC16 in *Ifnar*^−/−^ vs. WT mice. The lack of IFN-I signaling resulted in complete abrogation of the CD8^+^ T-cell response (Figures 5E,F).

### The formulation of OVA/CpG-ODN with COA-ASC16 enhances *in vivo* co-uptake of OVA and CpG-ODN by dendritic cells

**Figure 6:**
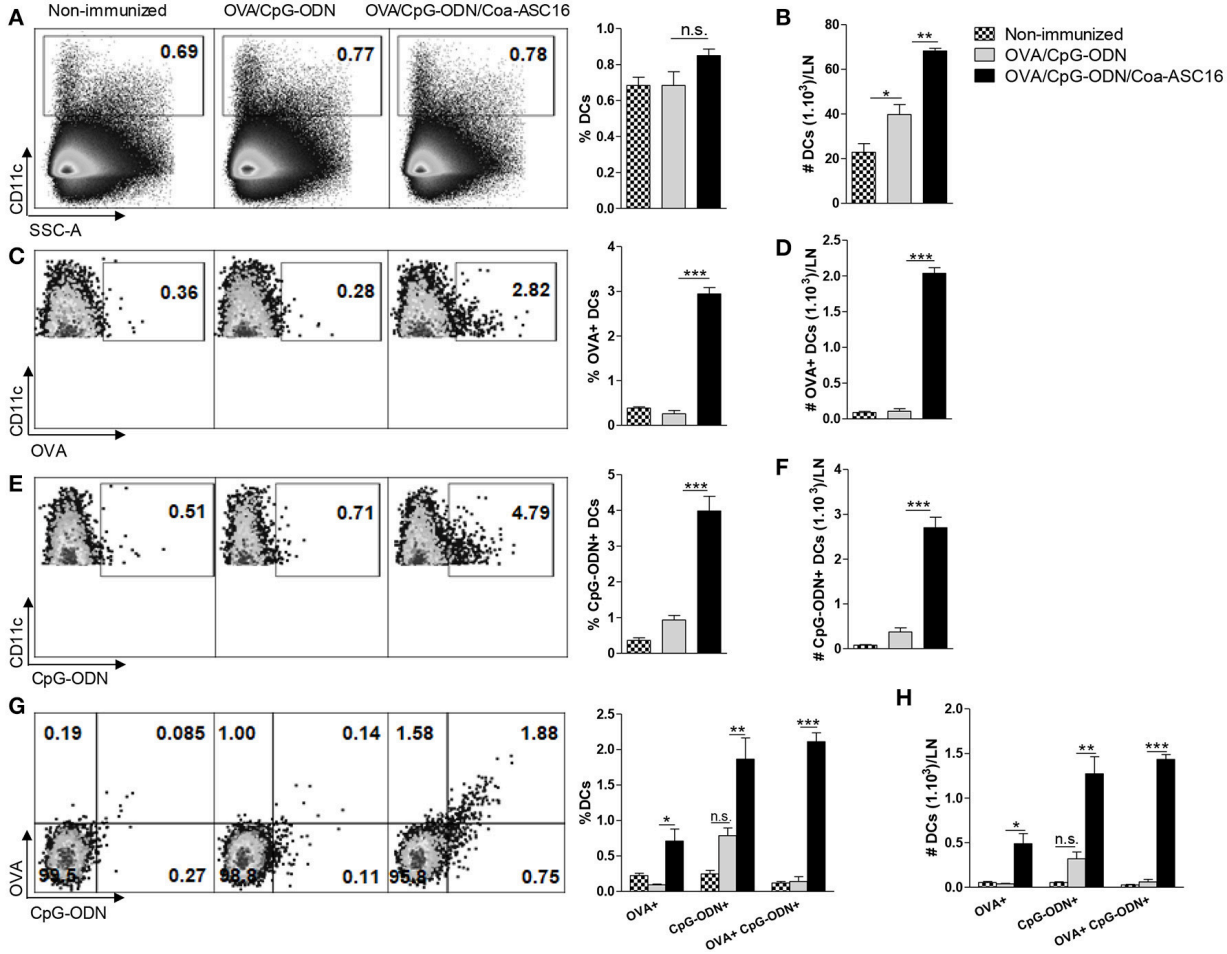
The formulation of OVA/CpG-ODN with Coa-ASC16 enhances *in vivo* co-uptake of OVA and CpG-ODN by DCs. C57BL/6 mice were immunized in both hind limbs with Alexa Fluor 647-OVA/Alexa Fluor 488-CpG-ODN or Alexa Fluor 647-OVA/Alexa Fluor 488-CpG-ODN/Coa-ASC16. Seventy-two h later, cell suspension from inguinal LN were analyzed by flow cytometry. **(A)** Percentage and **(B)** absolute number of total CD11c^+^ DCs. **(C)** Percentage and **(D)** absolute number of total OVA^+^ CD11c^+^ DCs. **(E)** Percentage and **(F)** absolute number of total CpG-ODN^+^ CD11c^+^ DCs. **(G)** Percentage and **(H)** absolute number of OVA^+^ or CpG-ODN^+^ simple positive and OVA^+^ CpG-ODN^+^ double positive within CD11c^+^ DCs. The data show the mean ± SEM of individual values (3-4 mice/treatment group in each experiment) and are representative of two independent experiments performed. n.s., not significant, ^*^p < 0.05, ^**^p < 0.01, ^***^p < 0.001.

Targeting antigen and adjuvant at the same antigen presenting cell generally results in a potent induction of effector T-cells and therefore is an attractive strategy for vaccine development (36). Considering the robust CD8^+^ T-cell response displayed by our adjuvant strategy we speculated that the nanostructured formulation could promote the uptake of OVA and CpG-ODN by dendritic cells (DCs). To this aim, draining LN were collected 72 h after immunization with the different formulations. Mice immunized with OVA/CpG-ODN/Coa-ASC16 showed the highest total number of CD11c^+^ cells (Figures 6A,B). These cells were indeed characterized by an enhanced engulfing of both OVA and CpG-ODN in comparison to cells from mice immunized with OVA/CpG-ODN (Figures 6C–F). In addition, despite the fact that our system does not chemically link OVA to CpG-ODN, mice immunized with OVA/CpG-ODN/Coa-ASC16 were able to simultaneously load both molecules to the same CD11c^+^ cells more efficiently than mice immunized with OVA/CpG-ODN (Figures 6G,H).

### The formulation of OVA/CpG-ODN with Coa-ASC16 enhances expansion and polyfunctionality of effector CD8^+^ T-cells

**Figure 7:**
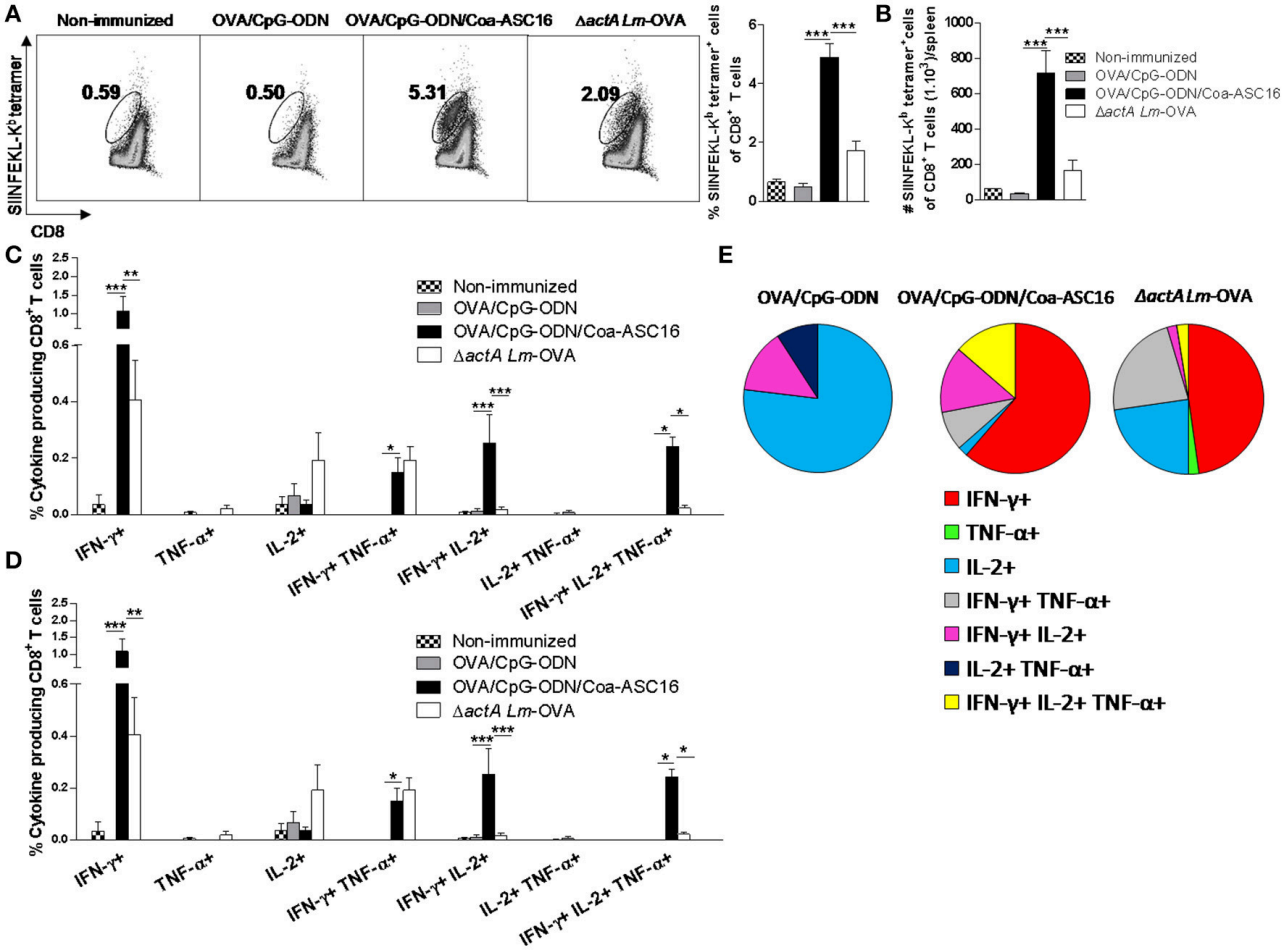
The formulation of OVA/CpG-ODN with Coa-ASC16 enhances expansion and polyfunctionality of effector CD8^+^ T-cells. C57BL/6 mice were immunized on day 0 with OVA/CpG-ODN, OVA/CpG-ODN/Coa-ASC16 or Δ*actA Lm-*OVA and euthanized on day 7 for spleen extraction. **(A)** Percentage of SIINFEKL-K^b^ tetramer^+^ CD8^+^ T-cells. **(B)** Total number of SIINFEKL-K^b^ tetramer^+^ CD8^+^ T-cells/spleen. **(C–E)** CD8^+^ T-cell cytokine production from splenocytes after *in vitro* stimulation with SIINFEKL peptide. Multiparameter flow cytometry was used to determine **(C)** percentage and **(D)** total number of cytokine producing CD8^+^ T-cells expressing each of the seven possible combinations of IFN-γ, TNF-α, and IL-2, **(E)** fraction of total CD8^+^ T-cell response comprising cells expressing each of the seven combinations of IFN- γ, TNF-α, and IL-2. For gating strategy and representative flow cytometry data, see Supplementary Figure 3. The data show the mean ± SEM of individual values (4 mice/treatment group in each experiment) and are representative of two independent experiments performed. ^*^p < 0.05, ^**^p < 0.01, ^***^p < 0.001.

The *in vivo* cytotoxicity assay revealed a robust lysis rate against target cells loaded with SIINFEKL peptide in mice vaccinated with nanostructured formulation. Here, we analyzed the expansion of SIINFEKL-specific CD8^+^ T-cells by staining with SIINFEKL-Kb tetramer and the number of CD8^+^ T-cells producing simultaneously cytokines (polyfunctionality) in different experimental groups. The antigen-specific CD8^+^ T-cell responses induced by the non-replicative vaccines (OVA/CpG-ODN and OVA/CpG-ODN/Coa-ASC16) was compared with that induced by the attenuated, but replicative competent live vaccine, Δ*actA Lm*-OVA. Mice immunized with OVA/CpG-ODN/Coa-ASC16 presented a higher expansion of SIINFEKL-K^b^ tetramer^+^ CD8^+^ T-cells than mice immunized with the other vaccine models (Figures 7A,B). To analyze the quality of the CD8^+^ T-cell response, we performed an intracellular staining of IFN-γ, TNF-α, and IL-2. Immunization with OVA/CpG-ODN/Coa-ASC16 showed a higher percentage and total number of (IFN-γ^+^IL-2^+^TNF-α^+^) triple, (IFN-γ^+^IL-2^+^) double, and (IFN-γ^+^) single positive CD8^+^ T-cells than the other immunization groups (Figures 7C–E, Supplementary Figure 3 for gating strategy used). Therefore, our results show that vaccination with nanostructured formulation cause the highest expansion of polyfunctional CD8^+^ T-cells, which would correlate with protection against intracellular infection.

### CD8^+^ T-cell response induced by OVA/CpG-ODN/Coa-ASC16 protects against *Lm*-OVA infection

**Figure 8:**
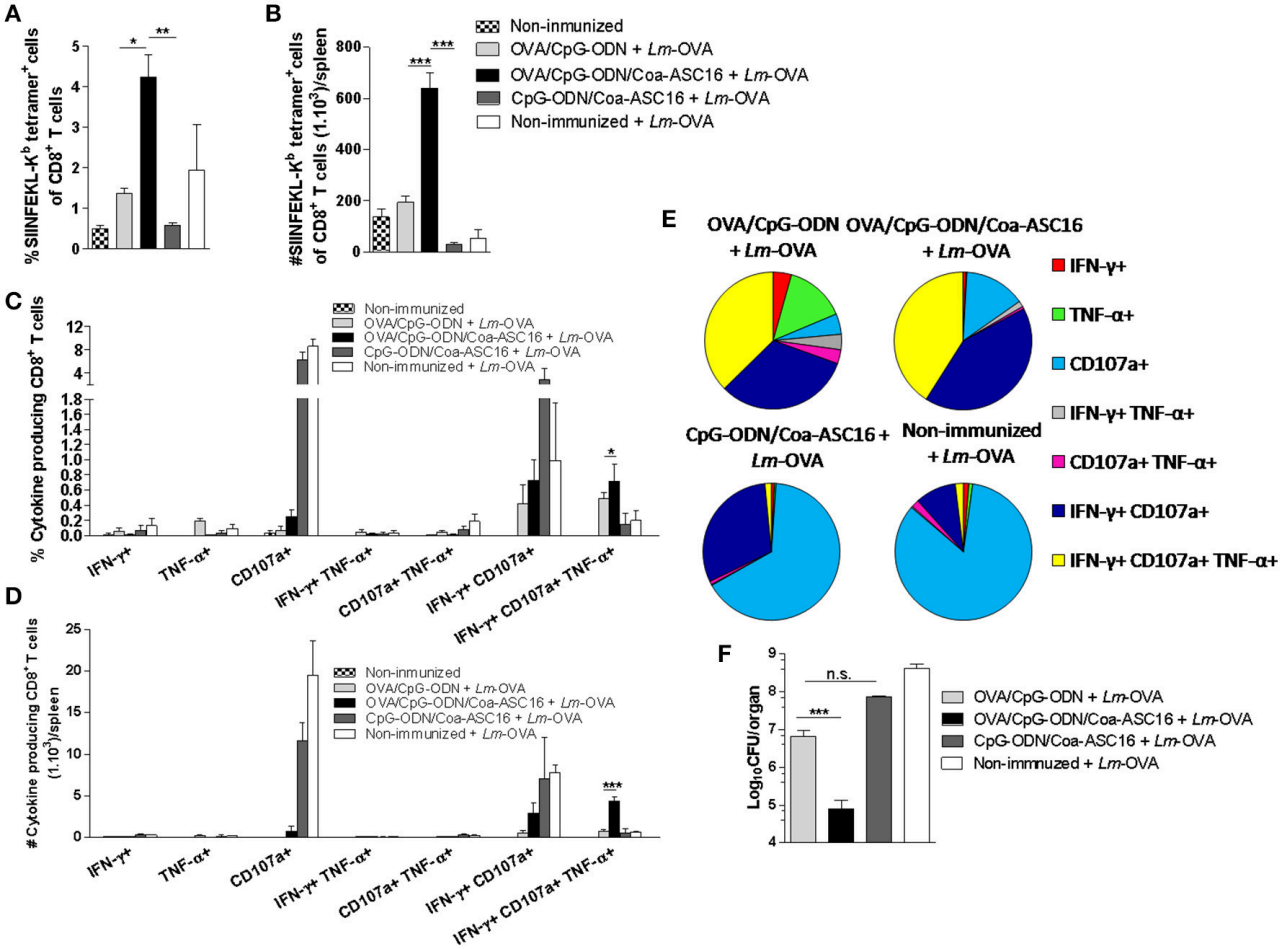
CD8^+^ T-cell response induced by the nanostructured formulation protects against *Lm*-OVA infection. C57BL/6 mice were immunized on day 0 with OVA/CpG-ODN, OVA/CpG-ODN/Coa-ASC16 or CpG-ODN/Coa-ASC16. Seven days later, immunized and non-immunized mice were intravenously infected with 1 × 10^5^ CFU of *Lm-*OVA. Two days after infection, mice were euthanized for liver and spleen extraction. **(A)** Percentage of SIINFEKL-K^b^ tetramer^+^ CD8^+^ T-cells in spleen. **(B)** Total number of SIINFEKL-K^b^ tetramer^+^ CD8^+^ T-cells/spleen. **(C–E)** CD8^+^ T-cell cytokine production from splenocytes after *in vitro* stimulation with SIINFEKL peptide. Multiparameter flow cytometry was used to determine **(C)** percentage and **(D)** total number of CD8^+^ T-cells expressing each of the seven possible combinations of IFN-γ, TNF-α, and CD107a, **(E)** the fraction of the total CD8^+^ T-cell response comprising cells expressing each of the seven combinations of IFN-γ, TNF-α and CD107a. For gating strategy and representative flow cytometry data, see Supplementary Figure 4. **(F)** Bacterial load in liver. The data show the mean ± SEM of individual values (3–4 mice/treatment group in each experiment) and are representative of two independent experiments performed. n.s., not significant, ^*^p < 0.05, ^**^p < 0.01, ^***^p < 0.001.

To test the protective capacity of OVA-specific CD8^+^ T-cell response induced by the nanostructured formulation, we challenged immunized mice with *Lm*-OVA and analyzed the CD8^+^ T-cell response in spleen and the remaining bacterial CFU burden in liver. In correlation with the superior expansion of antigen-specific CD8^+^ T-cells (Figures 8A,B), mice immunized with OVA/CpG-ODN/Coa-ASC16 induced a higher amount of IFN-γ^+^CD107a^+^TNF-α^+^ CD8^+^ T-cells than mice immunized with OVA/CpG-ODN (Figures 8C–E, Supplementary Figure 4 show gating strategy used). In concordance with these results, mice vaccinated with nanostructured formulation were able to remarkably reduce the bacterial load in liver. Two days after infection, high bacterial loads were recovered in the liver of mice vaccinated with OVA/CpG-ODN, while OVA/CpG-ODN/Coa-ASC16-immune mice had reduced this bacterial burden by ~2 logs (Figure 8F).

## Discussion

We formulated CpG-ODN with a novel nanostructure with the aim to optimize CpG-ODN adjuvant activity and used OVA as soluble protein antigen model. Our previous work proved that this system is an efficient strategy to elicit strong antibodies (IgG1 and IgG2a) and Th1/Th17 cellular responses in mice with a three-dose immunization regimen and without toxic systemic effects (16). However, the mechanisms whereby Coa-AS16 improves antigen-specific immune response are still not fully elucidated. Based on their mechanisms of action, vaccine adjuvants are classified as immunostimulatory agents, depot systems or vehicles (2, 4). In this regard, we have previously shown that Coa-ASC16 promotes antigen retention at the injection site (depot effect) and is sensed by the innate immune system promoting a transient local inflammatory environment involving the release of Damage-Associated Molecular Patterns, cytokines (IL-1β, IL-6, and IL-12) and recruitment of innate cells (neutrophils and Ly6C^high^ monocytes) (31). These facts indicate Coa-ASC16 acts as an antigen reservoir and immunostimulatory agent. In this study, we demonstrate the ability of this adjuvant strategy to elicit an additional effector CD8^+^ T-cell response while allowing the reduction of antigen-dose and immunization doses without significantly compromising the antigen-specific immune response.

The use of Coa-ASC16 to formulate OVA/CpG-ODN allows the induction of a long-lasting antibody response with a single-dose avoiding the need of boosts, a goal desired in prophylactic vaccines (Figure 4). Additionally, the nanostructure improves the antibody avidity (Figure 2F) up to a level comparable to the one reached by using the gold standard CFA adjuvant but also the most reactogenic of known adjuvants and hence is unsuitable for human use. Previous studies demonstrated that vaccines that have the ability to improve antibody response have superior capacity to induce germinal center formation (37–39), unique lymphoid microenvironment in which antigen-activated B cells undergo class switching, affinity maturation, and differentiation into memory B cells. These studies suggest than the nanostructured formulation could modify several mechanisms essential for germinal center formation, a potential linkage that will be important to investigate in subsequent studies.

CpG-ODN is able to induce CD8^+^ T-cell immunity only under certain conditions. CpG-ODN is not optimally effective to elicit this type of response when is used in a soluble format. However, CpG-ODN is able to induce CD8^+^ T-cell immunity when it is conjugate with antigen (40) or formulated with different strategy. Here, we report that the formulation of antigen/CpG-ODN (both molecules without conjugation) with Coa-ASC16 improve its ability for inducing CD8^+^ T-cell immunity comparable to other strategies. However, it is difficult to compare different formulations of CpG-ODN reported in the literature because the amount and the type of CpG ODN and antigen as well as mouse strain are divergent. For example, cytotoxic activity of CD8^+^ T-cells induced by CpG-ODN/Coa-ASC16 with a single-dose (Figure 5) is similar to that induced after a single immunization with CpG-ODN formulated with a nanoemulsion (41) or CpG-ODN nanoparticulated (42) or after two immunizations with CpG-ODN conjugated with an albumin-binding lipid (43). The formulation with Coa-ASC16 has the advantage that is a simple and inexpensive platform.

Regarding the powerful CD8^+^ T-cell response observed by a single immunization with OVA/CpG-ODN/Coa-ASC16 (Figure 5), we attributed this phenomenon to two main factors. First, the nanostructured formulation clearly elicits a higher antigen-specific CD8^+^ T-cell expansion than the other formulations including the live vaccine vector, Δ*actA*-*Lm*-OVA (Figures 7A,B). Second, the characterization of this response revealed a high degree of polyfunctionality including IFN-γ^+^TNF-α^+^IL-2^+^ triple positive CD8^+^ T-cells (Figures 7C–E), which often correlates with better protection against infection. For example, this phenomenon was previously demonstrated in mice vaccinated against *T. cruzi* using adenovirus vector (44), and in mice vaccinated against malaria with adenoviral and modified vaccinia virus Ankara vectors (45). In addition, human polyfunctional CD8^+^ T-cells correlate with protection in HIV and *Mycobacterium tuberculosis* infection (46–49). Consistently with these findings, antigen-specific effector CD8^+^ T-cells induced by OVA/CpG-ODN/Coa-ASC16, after challenge with *Lm*-OVA, showed higher efficiency to combat this bacterial intracellular infection than the other immunization strategies (Figure 8F). Additionally, it has been described that polyfunctional IFN-γ^+^TNF-α^+^IL-2^+^ CD8^+^ T-cells have higher capacity for surviving and providing greater memory protection compared with those that produce only one cytokine (50, 51). Therefore, the high number of polyfunctional CD8^+^ T-cells observed in mice immunized with OVA/CpG-ODN/Coa-ASC16 reveals the potential of this adjuvant strategy for developing memory CD8^+^ T-cell immunity, the ultimate goal for vaccines. This issue is currently under investigation by our group.

Seeking to explore in more detail the mechanisms by which the Coa-ASC16 improves the antigen-specific immune response, we focused on IL-6. This cytokine has been described as a promoter of the T-follicular helper cells differentiation and germinal center activation (24) and hence it has been reported as a key player for enhancing humoral response in many adjuvant strategies (25–30). Despite that we have previously reported local high levels of IL-6 released in response to Coa-ASC16 injection (31), the lack of this cytokine had no effect in the enhancer of magnitude or antibody isotype switching of the humoral response elicited by the nanostructured formulation with a three-dose schedule (Figure 2E). Yet, its possible role on inducing an earlier seroconversion or somatic hypermutation within the germinal center needs further elucidation. Moreover, the role of IL-6 on the improvement of humoral response *in vivo* is still controversial. It has been shown that although IL-6 may play a significant role during early stages of the immune response, its impact on late stages is low (52, 53). Other reports even suggest that in certain models, the absence of IL-6 can be compensated by other cytokines such as IL-21 and IL-27 (24). In relation to CD8^+^ T-cell response, several reports showed that IL-6 plays a role in the promotion of effector response in viral infections (54) or in vaccination using monophosphoryl lipid A/alum as adjuvant (22). In contrast, we found that IL-6 is not necessary to induce CD8^+^ T-cell response by OVA/CpG-ODN/Coa-ASC16 (Figures 2C,D). Collectively, the considerable variation among the molecular mechanisms found to be involved in different adjuvant systems, highlights the importance of studying each individual system separately.

Although the complete mechanism by which this nanostructured formulation works is yet unknown, we speculate that the slow antigen release (31) is the main reason why we observed a similar long-lasting humoral and an effector CD8^+^ T-cell response after reducing the number of immunizations from three doses to one (Figures 4, 5A). A similar outcome is observed when reducing to one third the antigen dose, demonstrating the Coa-ASC16 ability for dose-sparing (Figure 3).

It is important that our vaccine model mimics the composition of a subunit vaccine (based on highly purified or recombinant antigens). In order for this kind of vaccine to elicit a CD8^+^ T-cell response, the nature of antigen (particulate/non-particulate, conjugate/unconjugated with adjuvant) and the inflammatory environment in which the DCs encounter the antigen are extremely important. A proper inflammatory environment at the injection site and draining LN provide the signals necessary for activation/maturation of DCs and the nature of the antigen impact on the efficiency of antigen uptake and presentation by DCs (55). Particularly, for an exogenous antigen like OVA to be presented by DCs to naïve CD8^+^ T-cells, it must undergo a process called cross-presentation (56). This process, in addition to antigen cytosolic delivery into DCs, require co-association of signal of an appropriate adjuvant to the same DCs (36). In mice, resident CD8^+^ DCs and migratory CD103^+^ DCs have been described as the most efficient cross-presenting DCs. Moreover, pDCs has been shown to efficiently cross-present when stimulated by TLR ligands (57). Cross-presentation can be facilitated by signals deployed by intracellular Toll-like receptors agonists like CpG-ODN and/or cytokines like IFN-I, among others (34, 35, 58, 59). To secure the co-localization of both molecules within the same DCs, several adjuvants strategies involving CpG-ODN used chemical or physical conjugations between the antigen and the CpG-ODN (40, 60). In this work, we found that the nanostructured formulation is able to induce a strong CD8^+^ T-cell response and that requires both, CpG-ODN and IFN-I signaling. This is proved by our findings where immunization with OVA/Coa-ASC16 or the abrogation of IFN-I pathway completely impairs CD8^+^ T-cell response (Figures 2A,B, 5E,F). The CpG-ODN-dependency for exerting a CD8^+^ T-cell response suggests that the co-localization of OVA and CpG-ODN by the same DCs is important for its licensing and posterior priming of naïve CD8^+^ T-cells. In this regard, we have shown that our adjuvant strategy promotes the co-uptake of OVA and CpG-ODN by DCs in draining LN (Figure 6G,H) despite being offered in an unconjugated manner (Figure 1C). These results, combined with the efficacy of CD8^+^ T-cell response, invite us to elucidate which subset of DCs could be involved in this process and to understand how this nanostructure promotes co-uptake of both molecules. The characterization of this phenomenon will allow us to confirm if there is a strict link between uptake, cross-presentation and the optimizing of activation of CD8^+^ T-cell (cross-priming). Although these inquiries remain unknown, we hope to address them on the near future.

Focusing on IFN-I, it is still unclear whether its production is being elicited by the CpG-ODN or/and the Coa-ASC16. Nevertheless, this fact is specially intriguing since we used a type of CpG-ODN that belongs to class-B family. As a consequence of its structure, class-B CpG-ODN are not characterized for inducing IFN-I as other CpG-ODN families such as class-A CpG-ODN (13). A possible mechanism involved in the induction of these cytokines might be related to the Coa-ASC16 itself. It has been reported that self or pathogen dsDNA can activate cytosolic DNA sensor proteins inducing the production of IFN-I (61). Considering Coa-ASC16 ability for inducing *in vivo* death of resident cells at the injection site with the transient release of Damage-Associated Molecular Patterns as dsDNA previously shown (31), this nanostructure might be triggering the secretion of IFN-I by the activation of cytosolic DNA sensor proteins. Yet, the fact that self dsDNA could additionally activate Toll-like receptor 9 pathway should not be dismissed. Here, we present an adjuvant strategy that efficiently induced IFN-I dependent CD8^+^ T-cell response using class-B CpG-ODN not conjugated with the antigen. This is a desired effect for two main reasons. First, free class-A CpG-ODN aggregates into uncontrolled higher order structures of different sizes that are unpredictable and hence not suitable for clinical applications (13). Second, the conjugation of a protein with CpG-ODN can disrupt the structure of the antigen affecting its properties (62).

In summary, these data indicate that the formulation system presented herein strongly optimizes CpG-ODN adjuvant activity thus enhancing quantitatively and qualitatively both humoral and CD8^+^ T-cell responses with a single-dose, thus providing a substantive vaccine platform for use with subunit antigens. In addition, this system offers most of the desired effects in a vaccine adjuvant such as allowing the reduction of the number of immunizations and dose-sparing without compromising the resulting immune response while offering a simple, biocompatible, and inexpensive system that could easily scale up to massive production.

## Author contributions

AC and BM designed the experiments. AC performed most of the experiments, analyzed data, prepared figures, and collaborated in manuscript writing. MS, JD, MC, CM, SS, DA, SP, and MP-P and GM contributed to study design, analysis of results, and corrected the manuscript. BM conceived and supervised the study and wrote the manuscript.

### Conflict of interest statement

The authors declare that the research was conducted in the absence of any commercial or financial relationships that could be construed as a potential conflict of interest.

## Abbreviations

Δ*actA Lm-*OVA: attenuated *Listeria monocytogenes* 10403s strain with OVA construct
ASC16: 6-O-ascorbyl palmitate ester
CFA: Complete Freund's Adjuvant
CFU: Colony Forming Unit
Coa-ASC16 or coagel: nanostructure
CpG-ODN: synthetic oligodeoxynucleotides containing unmethylated cytosine-guanine motifs
DCs: dendritic cells
IFN-I: type I interferons
KSCN: potassium thiocyanate
*Lm-*OVA: *Listeria monocytogenes* 10403s strain with OVA construct
LN: lymph nodes
OD: optical density
OVA: ovalbumin
SIINFEKL: H-2K^b^ restricted OVA_257−264_
WT: wild-type.
