# Supplementary material for: Class-B CpG-ODN Formulated With a Nanostructure Induces Type I Interferons-Dependent and CD4+ T Cell-Independent CD8+ T-Cell Response Against Unconjugated Protein Antigen
Source: Front Immunol. 2018 Oct 10;9:2319. doi: 10.3389/fimmu.2018.02319 (PMC6192457; doi:10.3389/fimmu.2018.02319)
Supplement: Supplementary file 1 [file Data_Sheet_1.PDF]

Supplementary figures

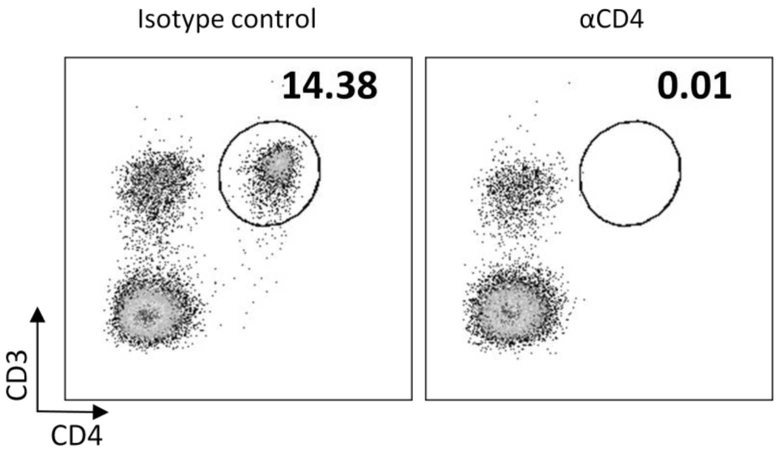

Supplementary figure 1. **Control of depletion of CD4+ T cells at the moment of the immunization.** Dot plots showing percentages of $CD4^+$ T cells in blood of mice immunized with OVA/CpG-ODN/Coa-ASC16 (day 0) treated on days -2, -1, 0 and 2 with anti-CD4 antibody or isotype control (IgG).

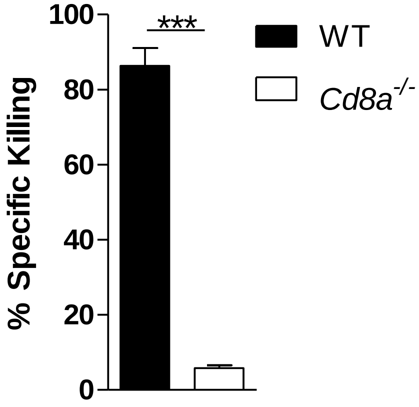

Supplementary Figure 2. The cytotoxic activity induced by the nanostructured formulation is exclusively mediated by $CD8^+$ T-cells. WT or *Cd8a*$^{-/-}$ mice were immunized at day 0 with OVA/CpG-ODN/Coa-ASC16. Bar graphs show percentage of specific lysis in an *in vivo* killing assay determined seven days post immunization. The data show the mean ± SEM of individual values (3-4 mice/treatment group in each experiment) and are representative of two independent experiments performed. ***p<0.001.

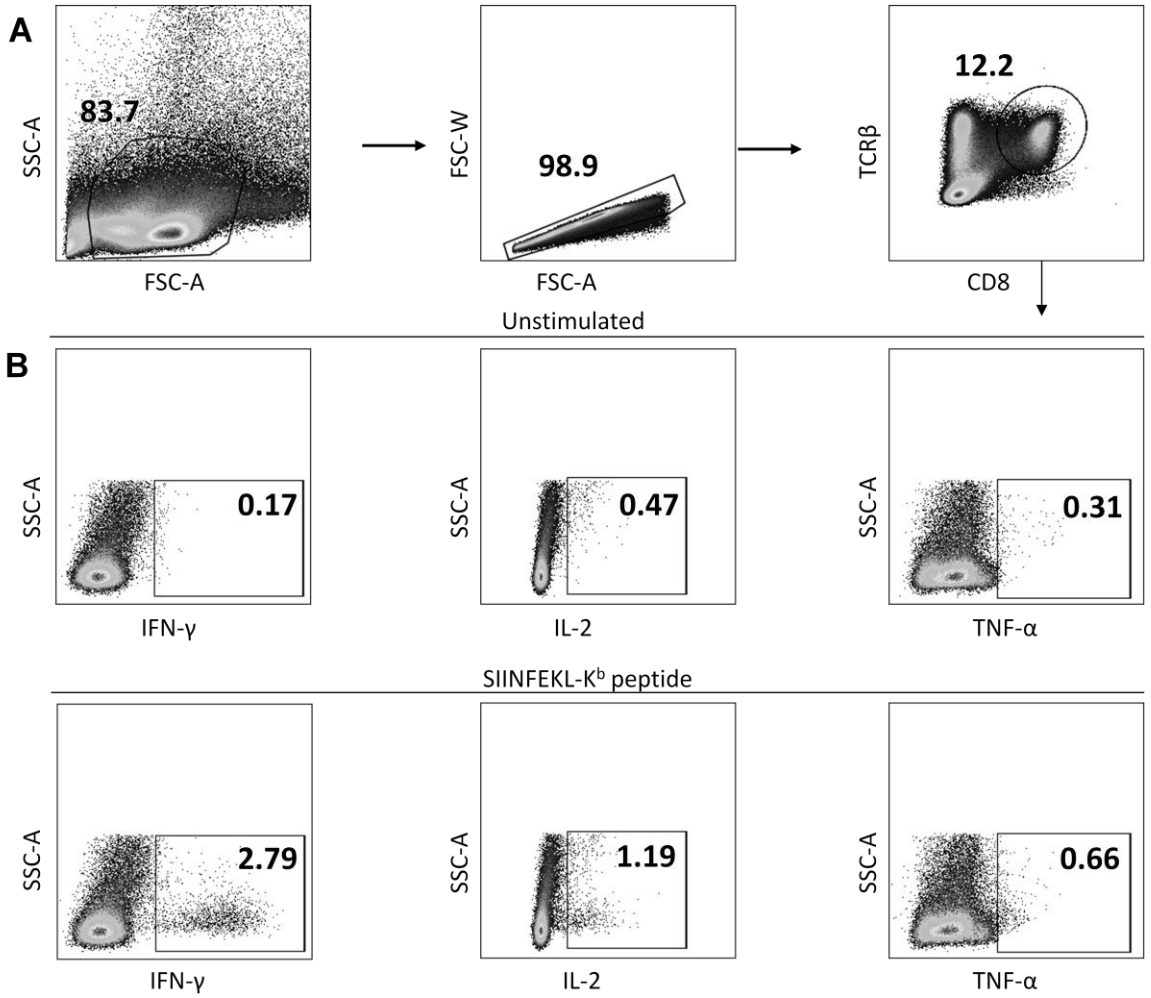

Supplementary Figure 3. Gating strategy used in Figure 5. (A) Selection of population containing $CD8^+$ T-cells. Each subsequent panel shows only the population of interest that has been selected from the gate on previous plot. **(B)** Selection of $CD8^+$ T-cells producing the cytokines of interest shown in an unstimulated and a SIINFEKL peptide stimulated sample from one individual. Boolean gating was used to calculate proportions of polyfunctional T cells.

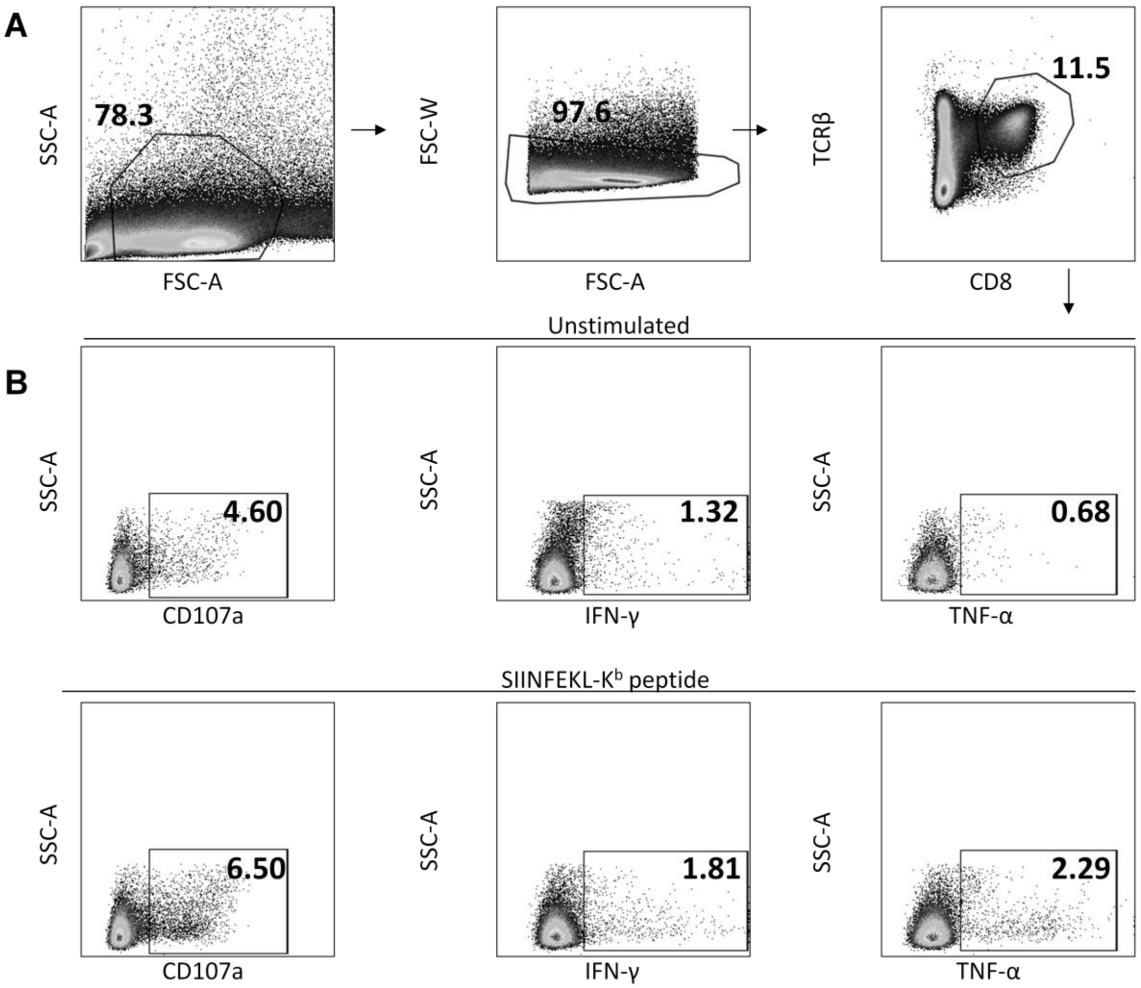

Supplementary Figure 4. Gating strategy used in Figure 6. (A) Selection of population containing $CD8^+$ T-cells. Each subsequent panel shows only the population of interest that has been selected from the gate on previous plot. **(B)** Selection of $CD8^+$ T-cells producing the cytokines of interest shown in an unstimulated and a SIINFEKL peptide stimulated sample from one individual. Boolean gating was used to calculate proportions of polyfunctional T cells.
